# Diagnosis and Treatment of Childhood Pulmonary Tuberculosis: A Cross-Sectional Study of Practices among Paediatricians in Private Sector, Mumbai

**DOI:** 10.1155/2015/960131

**Published:** 2015-08-26

**Authors:** Carolyn Kavita Tauro, Nilesh Chandrakant Gawde

**Affiliations:** ^1^School of Health Systems Studies, Tata Institute of Social Sciences, Sion-Trombay Road, Deonar, Mumbai, Maharashtra 400 088, India; ^2^Centre for Public Health, School of Health Systems Studies, Tata Institute of Social Sciences, Sion-Trombay Road, Deonar, Mumbai, Maharashtra 400 088, India

## Abstract

Majority of children with tuberculosis are treated in private sector in India with no available data on management practices. The study assessed diagnostic and treatment practices related to childhood pulmonary tuberculosis among paediatricians in Mumbai's private sector in comparison with International Standards for Tuberculosis Care (ISTC) 2009. In this cross-sectional study, 64 paediatricians from private sector filled self-administered questionnaires. Cough was reported as a symptom of childhood TB by 77.8% of respondents. 38.1% request sputum smear or culture for diagnosis and fewer (32.8%) use it for patients positive on chest radiographs and 32.8% induce sputum for those unable to produce it. Sputum negative TB suspect is always tested with X-ray or tuberculin skin test. 61.4% prescribe regimen as recommended in ISTC and all monitor progress to treatment clinically. Drug-resistance at beginning of treatment is suspected for child in contact with a drug-resistant patient (67.7%) and with prior history of antitubercular treatment (12.9%). About half of them (48%) request drug-resistance test for rifampicin in case of nonresponse after two to three months of therapy and regimen prescribed by 41.7% for multidrug-resistant TB was as per ISTC. The study highlights inappropriate diagnostic and treatment practices for managing childhood pulmonary TB among paediatricians in private sector.

## 1. Introduction

With estimated 80,000 deaths among children in 2013, tuberculosis (TB) continues to be major infectious disease among children [1]. Low- and middle-income countries bear disproportionate burden with more than 50% of the global cases occurring in Southeast Asia and the Western Pacific. India alone accounts for 27% of the global burden of paediatric tuberculosis [2] and nearly five percent of the new cases in India are reported among children [3]. Early detection and treatment are the key to prevent mortality and limit morbidity. It is equally valuable in prevention of multidrug-resistant tuberculosis (MDR-TB), which affects nearly 32000 children globally [4]. The private general practitioner is usually the first point of contact for patients in India [5]. Majority of TB patients receive care from a large variety of providers outside of the national TB programme despite the high costs and varying quality [6]. Even those registered by India's Revised National TB Control Programme (RNTCP) reach the programme after going through cycles of consultation with private practitioners (both qualified and nonqualified) [7]. Management of childhood TB in the private sector, especially adherence to standard treatment guidelines, is crucial for limiting effects of the disease among children.

The first study in India investigating management practices of TB in private sector revealed high magnitude of inappropriate prescribing practices [8]. Since then, many other studies have enquired into management practices in private sector. The studies highlight high variance in clinical practice and most of the practices being inappropriate ones in India [9–12]. Lack of knowledge of TB treatment regimen and inappropriate prescriptions have been reported across multiple countries [13–16]. In 2006, Tuberculosis Coalition for Technical Assistance (TBCTA) developed International Standards for Tuberculosis Care (ISTC) with the purpose of laying down a widely accepted level of care that all practitioners, public and private, should seek to achieve in managing patients who have, or are suspected of having, tuberculosis [17]. Since then, researchers had looked for compliance with these broad set of standards. Evidence from India shows that practices in private sector often deviate from the standards laid down in ISTC [18–20]. Most of these studies have focused on general practitioners (with no post graduate qualification in medicine) who manage tuberculosis among adults.

Compared with this, management of TB in children in private sector is still unexplored in India. Appropriate treatment of TB is essential to prevent death, drug-resistance, and adverse effects among affected children. It also will decrease risk of transmission to susceptible contacts. It is therefore pertinent to explore diagnostic and treatment practices in private sector and difficulties they face while providing care to children infected with TB. Many guidelines exist for tuberculosis diagnosis, treatment, and control globally and in India. The World Health Organisation (WHO) Guidance for national tuberculosis programmes on the management of TB in children, the Indian guidelines of the RNTCP, WHO's Rapid Advice-Treatment for tuberculosis in children, ISTC, and the Consensus Statement formed by the Indian Academy of Paediatrics (IAP) are but a few of these. While the first two are more applicable to national health programme that presume it to be the end provider, ISTC and IAP guidelines are more relevant for the private sector that manages TB in its own setting. The healthcare providers in private sector range from nonqualified medical practitioners, pharmacists, and qualified practitioners without training in paediatrics to paediatricians. There is, however, increasing tendency to seek care from paediatricians for a sick child in the city of Mumbai. Previous studies have documented practices among general physicians, but none among paediatricians. This study was conducted among paediatricians in Mumbai who treat TB patients with objective of comparing practices in diagnosis and treatment of TB and drug-resistant tuberculosis to standards set in the ISTC (2009).

## 2. Methodology

### 2.1. Study Settings

Mumbai is the second largest urban agglomeration in India with a population of over 18 million, more than 1.7 million of whom are children under the age of six [21]. Those who dwell in Mumbai face various environmental hazards including inadequate water and sanitation, pollution, and poor ventilation with almost half the population living in slums where many families live in a single room, making them vulnerable to diseases such as tuberculosis. At least 60,000 cases of TB are estimated to occur annually in the city [22]. A diverse healthcare system exists with both public and private sectors and has a range of healthcare service providers including traditional healers, nonqualified medical practitioners, qualified medical practitioners in primary care, and specialists who provide specific services and includes paediatricians. Public sector follows standardised treatment under India's RNTCP. Allopathy, Ayurveda, and Homeopathy are the main medical disciplines that are offered to patients. While most primary care providers (also referred to as family physicians) practice at single or two locations, most specialists including paediatricians provide their services in multiple healthcare facilities which may include public hospitals, trust hospitals, private-for-profit hospitals, many small size hospitals, and clinics in private sector. Patients diagnosed with TB in private sector may either be treated in private sector itself (most common) or referred to public sector for further management. Participation in Public Private Partnership schemes is poor and nearly half of TB patients of Mumbai continue to take treatment in private sector [22].

### 2.2. Study Design

The study was descriptive, cross-sectional, and quantitative in nature. It was carried out from December 2012 to February 2014, with data collection period from May 2013 to October 2013.

### 2.3. Development of Study Tool

The study tool was a self-administered questionnaire, which was developed by the researcher. The researcher has graduate training in medicine and at time of developing study tool had more than two years of experience in managing health interventions in the field of TB and HIV through a nongovernmental organisation in India. The researcher was pursuing master's degree in public health at time of conducting this research. During preparation of the tool, the ISTC and IAP guidelines were referred to [23, 24]. Another researcher with postgraduate training in community medicine and five years of field experience in management of tuberculosis reviewed the questionnaire. After incorporating comments from second researcher, questionnaire was pilot tested by administering it to a private paediatrician working in a tertiary private hospital in Mumbai.

### 2.4. Measurement of Variables

ISTC (2009) includes 21 standards covering four broad areas, namely, standards for diagnosis, standards for treatment, standards for addressing HIV infection and other comorbid conditions, and standards for public health. Since the objective was to assess diagnostic and treatment practices of childhood pulmonary TB including drug-resistant TB among paediatricians, eight standards pertaining to diagnosis and treatment, with components relevant to the study, were selected as follows.

*International Standards for Tuberculosis Care (ISTC) for Diagnosis and Treatment of Tuberculosis (Source: ISTC 2009)*

*Standards for Diagnosis*

 Standard 1: all persons with otherwise unexplained productive cough lasting two-three weeks or more should be evaluated for tuberculosis.
 Standard 2: all patients, including children who are capable of producing sputum, suspected of having pulmonary tuberculosis should have at least 2 sputum specimens obtained for microscopic examination.
 Standard 4: all persons with chest radiographic findings suggestive of tuberculosis should have sputum specimens submitted for microbiological examination.
 Standard 6: children suspected with intrathoracic TB should have sputum examined (by expectoration, gastric washings, or induced sputum) for smear microscopy and culture. In children with negative sputum smears diagnosis should be based on the finding of chest radiographic abnormalities consistent with TB, a history of exposure to an infectious case or evidence of TB infection (positive TST or IGRA) and clinical findings.

*Standards for Treatment*

 Standard 8: all patients who have not been treated previously should receive an internationally accepted first-line treatment regimen using drugs of known bioavailability. The initial phase should consist of 2 months of Isoniazid, Rifampicin, Pyrazinamide, and Ethambutol and continuation phase consists of H and R for 4 months, with doses conforming to international recommendations. Fixed drug combinations (FDCs) are highly recommended.
 Standard 10: patients with pulmonary TB should be monitored for response to therapy by follow-up sputum microscopy (2 specimens) at the time of completion of initial phase of treatment (2 months). If smear positive at this stage, smears should be repeated at 3 months, and if positive, culture and DST should be performed. In children, response to treatment is best assessed clinically.
 Standard 11: patients should be assessed for DR-TB based on history of prior treatment, exposure to a possible source case of DR-TB, and community prevalence of DR. DST should be conducted at start of therapy for previously treated patients with assessment for DR-TB in all those who remain sputum smear positive at completion of 3 months of treatment and those who have failed, defaulted, or relapsed following one or more courses of treatment. Culture and DST plus resistance to at least isoniazid and rifampicin should be performed in patients in whom DR is likely.
 Standard 12: those likely to have TB caused by DR organisms should be treated with specialized regimens containing second-line antituberculosis drugs; regimen should be standardized or based on suspected or confirmed DS patterns. At least 4 drugs to which the organisms are known to be susceptible, including an injectable agent, should be used for at least 18–24 months beyond culture conversion.

For assessing compliance to these standards, suitable items and indicators were developed for each of the selected standards (Table 1). The indicators related to diagnosis included “cough listed as one of the symptoms that would lead to suspicion of TB,” advising sputum smear/culture for TB suspects, those with positive chest X-rays, advising gastric lavage or bronchoalveolar lavage if child is not able to bring out sputum, and whether chest X-ray and/or tuberculin skin test were advised along with sputum smear/culture. The indicators related to treatment included “whether regimen prescribed is two months of four drugs (isoniazid, rifampicin, pyrazinamide and ethambutol) followed by four months of two drugs” (isoniazid and rifampicin), “whether response was monitored clinically,” drug-resistance suspected at beginning of treatment in cases of “previous treatment with anti-tubercular drugs” and/or “history of contact with a known case of multidrug-resistant TB,” “whether those who are smear positive at three months are tested for sensitivity to rifampicin,” and whether the “regimen prescribed for MDR-TB includes four new drugs with one injectable and one fluoroquinolone.” Standard 11 states that culture and drug sensitivity tests with at least those for isoniazid and rifampicin should be performed for patients with drug-resistance. However, traditional cultures are associated with delay in diagnosis and rapid molecular tests like Xpert MTB/RIF are recommended by WHO. Since Xpert MTB/RIF does not involve culture and tests for only rifampicin resistance, we measured “testing for rifampicin resistance” without necessity of culture and resistance testing for isoniazid.

Information was also collected on relevant aspects of management of childhood pulmonary tuberculosis. These descriptive variables included paediatricians' characteristics including age, sex, duration of clinical practice, and average number of new TB patients treated every month and the guideline referred by them for managing TB in children. With respect to diagnosis, respondents were asked to list signs that may suggest tuberculosis and challenges faced (if any) in diagnosis. With respect to first regimen offered by paediatricians, we also asked about dosages of each drug prescribed, frequency of administration of drugs (daily or intermittent), and challenges in treatment of childhood pulmonary TB. For assessing practices related to monitoring progress of treatment, investigations advised during and at the end of treatment were enquired into. The questionnaire included a set of questions related to diagnosis of MDR-TB. Apart from the primary outcomes listed in previous paragraph, these included “usual duration of therapy after which non-response to treatment is first suspected” by the practitioner, “action taken for such patients,” “usual duration of therapy after which drug-resistance is suspected,” “basis of diagnosis of MDR-TB” (bacteriological, clinical, radiological, or combination of these), “type of bacteriological test advised to diagnose drug-resistance” (culture or molecular techniques or both), and “laboratory where the investigation is carried out” (accredited or not accredited). For patients diagnosed with drug-resistant TB, further line of management was enquired into (treat the patient or refer to another practitioner or health facility).

### 2.5. Participants

The inclusion criteria for participants were postgraduate qualification in paediatrics (allopathy stream) practicing in private sector in Mumbai and those who treated children with pulmonary tuberculosis.

An initial list of paediatricians was prepared by approaching Indian Academy of Paediatricians (IAP), online local directory websites, and names suggested by study participants. Paediatricians were approached in person at their respective healthcare facilities and at a conference or by e-mail. E-mail provided link to an open source website harbouring an online version of the questionnaire developed for the study. Gentle reminders were sent once a week for four weeks to the practitioners. Paediatricians who consented to participate were provided with either the hard copy or soft copy of the questionnaire.

### 2.6. Analysis of Data

The questionnaire included some closed ended questions (e.g., whether you treat or refer MDR-TB patients). Such data was precoded. Other items were open ended (e.g., list investigations advised when you suspect a child to be suffering from pulmonary TB). For open ended items, responses were listed and categorised (e.g., in line with ISTC or not) and codes were assigned. Data was entered in Microsoft Excel version 14.0.0 and analysis involved calculation of frequencies and percentages of practices for variables mentioned above. Although it was not primary aim of the study, the authors also referred to guidelines of IAP and compared the first regimen advised by the practitioners with IAP guidelines [24].

### 2.7. Ethical Considerations

Ethical norms were followed with an introduction to paediatricians about the nature and purpose of the study. Informed consent process highlighted confidentiality of respondents' identity and the filled questionnaires (hard copy) were kept under lock and key. The online filled questionnaires were accessible only to the researcher and access was protected by a password to the online account. The data entered in Excel file did not bear any personal identifiers. Doctors' doubts regarding funding by a pharmaceutical company were cleared with the assurance that the study was for academic purposes only and was self-funded with no attachments or conflict of interests. Written consent was taken and complete volunteerism was assured before collecting data. The doctors had choice of dissent at any point of answering the questionnaire or after. No incentives were offered for participation in the study.

## 3. Results

### 3.1. Profile of the Participants

A total of 644 doctors were approached: 197 (30.6%) in person and 447 (69.4%) by e-mail. The 197 paediatricians were reached by visits to seventeen tertiary hospitals, seven children hospitals, twelve small size hospitals, three polyclinics, 31 exclusive paediatric clinics, and one conference venue. Only 58 of the 197 (29.4%) participated in the study; response to e-mail questionnaire was poorer with six participants responding online. Participants' profile has been presented in Table 2. Two-thirds of participants were male and half were younger than 45 years. More than a third of paediatricians were treating three or more cases of childhood TB every month. Out of the 52 doctors who responded that they referred to a guideline, 17 (33%) referred only to guidelines of the IAP, while another 17 (33%) referred to IAP along with other guidelines, including 13 (25%) referring to IAP as well as RNTCP guidelines. 10 (19%) referred only to RNTCP guidelines. Other guidelines they had listed included that of the WHO and Center for Disease Control (CDC). None of the doctors reported referring to the ISTC guidelines.

### 3.2. Symptoms and Signs That Raise Suspicion of TB

Almost all paediatricians suspected tuberculosis in a child with fever and most suspected the disease in a child with cough of more than two-week duration or loss of weight (Table 3). Next common symptoms included child in contact with a case of TB and loss of appetite. Other symptoms (not shown in table) raising suspicion of TB included failure to thrive or no weight gain (reported by 19; 30%), swellings in the body (14; 22%), no improvement with general antibiotics (5; 7%), malaise (3; 4.5%), headache and convulsions (3; 4.5%), pain in abdomen (2; 3%), night sweats (2; 3%), postmeasles respiratory case (1; 1.5%), diarrhoea (1; 1.5%), and a history of residing in an overpopulated area (1; 1.5%).

Among signs, malnutrition, palpable matted lymph nodes, and respiratory sounds (crepitations and/or rhonchi) were reported by majority (Table 3). Less commonly reported signs included hepatomegaly (reported by 12; 19%), splenomegaly (11; 17%), neck stiffness (8; 12%), fever (7; 11%), clubbing (7; 11%), absence of a BCG scar (6; 9%), ascites (4; 6%), chronic sinus or ulcer (4; 6%), abdominal mass (2; 3%), and phlycten (2; 3%).

### 3.3. Investigations to Diagnose Pulmonary TB among Children

Almost all paediatricians reported asking for X-ray of chest for diagnosis of pulmonary TB when open ended question of “list investigations requested to diagnose pulmonary TB” was asked (Table 3). Majority paediatricians reported prescribing investigations including complete blood count (CBC), erythrocyte sedimentation rate (ESR), and tuberculin skin test (TST). More than one-third of the respondents reported advising sputum smears (and/or cultures) for presence of acid fast bacilli (AFB). Other investigations included immunoglobulins (reported by 7; 11%) and gamma interferon (3; 4.7%). All 24 paediatricians (38%) who advise sputum smear or culture also advise for chest X-ray and/or TST. Another 20 paediatricians (31.7%) reported using sputum test only for some patients; most commonly, test was requested for older children only. Among the 24 paediatricians who were prescribing sputum test for all TB suspects, 21 (87.5%) reported that they request inducing of sputum if child is not able to produce sputum sample. Equal number of paediatricians reported doing sputum examination for all patients whose X-ray findings were suggestive of tuberculosis. More commonly, TST was employed to confirm diagnosis (Table 3); the three others included Interferon Gamma Release Assay (IGRA), starting treatment without any further investigations and repeat X-ray after a course of antibiotics.

A total of 28 clinicians specified challenges faced while diagnosing childhood pulmonary TB. The challenges included patients not able to afford cost of diagnostic tests limiting options with paediatricians (reported by 10; 35.7%), absence of one specific test to confirm TB (9; 32.1%), certain diagnostic procedures like inducing sputum being difficult to implement in practice (7; 25%), and availability of certain tests limited to fewer laboratories (2; 7.1%).

### 3.4. Treating Pulmonary Tuberculosis among Children

Out of 64 private paediatricians, two doctors (3%) did not specify regimens prescribed by them. Regimens prescribed by remaining 62 practitioners have been presented in Table 4 and are compared with ISTC and IAP guidelines. All doctors were prescribing daily therapy. Regimens prescribed by 38 (61.4%) respondents were as per ISTC 2009 recommendations. However, other regimens prescribed were often in line with IAP recommendations. Almost all (57; 91.9%) paediatricians were following IAP recommended regimens. Table 4 also shows dosages of drugs prescribed by paediatricians in private sector. All respondents who provided information on drug dosages were prescribing ISTC recommended dosage for rifampicin (R) and ethambutol (E). But, doses of isoniazid (H) and pyrazinamide (Z) were often lower than ISTC recommendations (45% and 68%, resp.). Combining data on combination, duration, and dosages for all drugs (data on all three provided by 58 paediatricians), only ten (17.2%) practitioners out of 58 were prescribing appropriate regimen with dosages as recommended by ISTC 2009. When compared with IAP guidelines, dosages of almost all paediatricians were appropriate for R and H but nearly two-thirds of paediatricians were advising higher and lower dosage for E and Z, respectively.

A total of 37 paediatricians specified challenges faced by them with paediatric formulations in the treatment of children with TB. Doctors reported that young children found these tablets difficult to ingest (reported by 9; 24.3%) and the taste unpalatable (5; 13.5%). Other reported difficulties included different drug combinations making treatment confusing (8; 21.6%) unavailability of wider choice of drugs (6; 16.2%), adverse effects of drugs (1; 2.7%), and long treatment duration (1; 2.7%).

### 3.5. Monitoring Response to Treatment

All paediatricians were monitoring response to treatment clinically. Some paediatricians were not advising any routine follow-up investigation; 12.7% were not advising any investigation during treatment and 11.9% at end of treatment (Table 5). These investigations included those for assessing effectiveness of treatment (chest X-ray, sputum smear/culture, CBC, and ESR) as well as those for identifying adverse effects of drugs (liver and renal function tests). Chest X-ray was the most common investigation used for monitoring response during (61.9%) and at end of treatment (84.7%). Sputum smear or culture was requested by less than a third of respondents during treatment and only 8.5% were using it at end of treatment. Decision to stop treatment at end of full course of antitubercular drugs was a clinical one for all respondents, aided by X-ray in a third and sputum in 13.8% of cases.

### 3.6. Practices in Case of Nonresponse to Treatment and Diagnosing of Drug-Resistance

**Figure 1:**
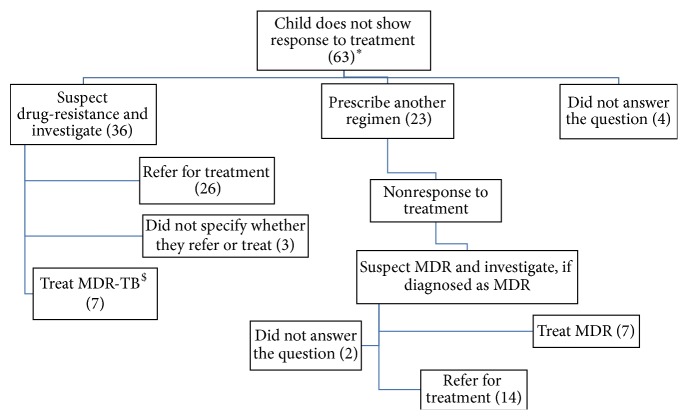
Actions taken by paediatricians when a patient does not respond to treatment. ^*∗*^one paediatrician reported that he never came across a patient who did not respond to treatment; figures in parenthesis are number of paediatricians following that practice, MDR (multidrug-resistant) TB; ^$^two of these seven paediatricians did not specify regimen.

If the patient was not improving clinically, paediatricians suspected that treatment is failing. Time at which nonresponse to treatment was suspected varied from practitioner to practitioner and was as less as one month to as long as 8 months but most (45 out of 50; 90%) were suspecting it early (within three months of therapy) (Table 6). Most common actions reported by 58 paediatricians included advising sputum culture and drug sensitivity test (35; 60.3%), suspecting and investigating for HIV infection (26; 44.8%), and prescribing another regimen (23; 39.7%). Since practices related to HIV-TB coinfection are not part of the paper, we have presented actions taken by paediatricians for HIV negative nonresponders in Figure 1. One paediatrician mentioned not coming across a nonresponder in practice. Out of the other remaining 63, four (6.3%) did not answer the question and 36 (57.1%) said that they investigate for MDR, whereas 23 (36.5%) mentioned that they prescribe another regimen (details of these regimens are provided subsequently). A total of 50 paediatricians answered question regarding time of suspecting drug-resistant TB, out of which 23 (46%) suspected only after trying second regimen (Table 6). Remaining 27 (54%) reported suspecting drug-resistance during first regimen itself, if sputum was positive at end of first (3; 11.1%), second (17; 63.0%), or third month (7; 25.9%) of therapy, respectively. Thus, 24 (48%) were suspecting drug-resistance at appropriate time (2 to 3 months), all of whom were testing for rifampicin resistance. A total of 31 paediatricians listed conditions when they suspected drug-resistance at beginning of treatment, two-thirds of them listed contact with case of drug-resistant TB, one-third investigated for resistance among HIV positive children, and only four (12.9%) reported suspecting resistance in cases treated with antitubercular drugs in the past. Almost all (48; 94.1%) paediatricians reported that basis of diagnosis of drug-resistance was bacteriological (Table 6). 33 of these who specified the type of test conducted reported use of sputum culture and drug sensitivity (reported by 10) or Xpert MTB/RIF (8) or both (15). Of the 35 who mentioned name of testing laboratories, 27 (77.1%) named laboratories that were accredited by national programme for drug-resistance testing whereas 8 (22.9%) were sending patients to nonaccredited laboratories.

### 3.7. Treatment for Nonresponders of Treatment

As stated previously and shown in Figure 1, 23 paediatricians did not investigate for drug-resistance when patient did not respond to first regimen; they chose to prescribe another regimen. Details of such second regimens offered prior to investigation of MDR-TB are presented in Table 7. It can be seen that 13 different regimens were prescribed by these 23 physicians. One or no drug was added by nine paediatricians (39%) whereas 14 (61%) reported adding at least two new drugs to previous regimen (eight adding two drugs, three adding three drugs, and four adding four drugs). Three of these 23 (4%) also reported having attempted a third regimen with addition of one new drug (one paediatrician) and two new drugs (two paediatricians) (not shown in table).

A total of 14 paediatricians reported treating drug-resistant TB and 12 of them (86%) provided details on the type of regimen. Of these 12 paediatricians, five (41.7%) were adding four new drugs including an injectable and a fluoroquinolone (Table 7). Another four (33.3%) said that drugs are chosen on basis of sensitivity test results but did not specify the number of drugs making it not possible to assess compliance with ISTC standards. The remaining three were not adding enough number of drugs or had no injectable drug in combination and were not as per ISTC standards.

## 4. Discussion

This cross-sectional survey reveals that diagnostic and treatment practices in management of childhood pulmonary tuberculosis among paediatricians in Mumbai's private sector deviate from practices recommended by the International Standards for Tuberculosis Care (ISTC) 2009. We discuss such deviations in detail and possible reasons of such inappropriate practice and its implications for tuberculosis control in Mumbai. Some studies earlier have assessed management practices for childhood TB and found them to be poor. Of these, one study assessed practices related to chemoprophylaxis in South Africa [25], another assessing appropriateness of regimens offered in public sector [26–28]. This is the first study from India that shows that management of childhood TB disease in private sector is also deviating from guidelines.

Prompt diagnosis of TB is one of the basic principles in management of this disease. In present study, more than a fifth of paediatricians did not mention cough as symptom of TB (Standard 1 of ISTC). This is likely to delay diagnosis and subsequently treatment with poor outcomes. Singla et al. [10] suggested that not all patients with respiratory symptoms receive an adequate evaluation for tuberculosis highlighting also the missed opportunities for earlier detection that could lead to an increased likelihood that TB bacilli will spread among family members and other people in the community. In previous studies of diagnostic practices, chest X-rays have been the commonest method used [10, 19, 20, 29, 30] and this study showed that it holds true for childhood TB as well. Few paediatricians reported using sputum smear or culture for diagnosis in young children who cannot cough up sputum easily (Standard 2) along with difficulty of implementing procedures like gastric lavage and bronchoalveolar lavages; also mentioned by previous investigators [30, 31]. In contrast to this, a recent study in the United Kingdom (UK), however, reported that about 77% of respondents use sputum induction for microbiological confirmation [32]. Several systematic reviews questioning reliability of serodiagnostic tests led WHO to release a negative policy recommending nonuse of such diagnostics, leading to its ban in India in 2012 [33, 34]. However few paediatricians in this study reported using these techniques in 2013, with possibility of serious epidemiological consequences.

Adequate and complete treatment of TB is cornerstone of TB control as it results in better health outcomes for affected patient as well as reduced chances of infection to others. Previous literature on treatment practices exhibits wide variance in practices, with almost every participating physician having a unique regimen to deal with TB [8, 10–12, 29]. Although less variance was found in first regimen offered by paediatricians compared to previous literature, more than one-third of paediatricians were following ISTC recommended regimen. While diagnosing TB in a child may be faced with practical difficulty as discussed already, there is no such hindrance for prescribing appropriate treatment as shown in a recent study in UK where adherence to ISTC recommended regimen was universal [32]. Appropriate combination and duration of drugs are not adequate if the dosages are incorrect. Many paediatricians prescribed lower doses of Isoniazid (H) and Pyrazinamide (Z) than recommended. Such lower dosage may potentiate drug-resistance especially when there are no mechanisms to ensure adherence to treatment. Treatment is only prescribed in private sector; there is no mechanism to supervise patients [8, 12]. It is also evident that adherence to treatment is often low in private sector [29]. Mechanisms to ensure adherence for patients treated in private sector are a priority and need operational research including piloting of interventions.

Drug-resistant TB is a bacteriological diagnosis for which sputum culture is considered to be the gold standard. However, in this study, many practitioners reported the use of clinical and radiological, and in a few cases serological, means to diagnose DR-TB [23]. This could on the one hand imply missing some children with DR-TB not investigated bacteriologically, while on the other hand other children may unnecessarily be on second- and third-line regimens without bacteriological confirmation. In this study, less than half who advised bacteriological diagnosis asked for both rapid and culture tests; some used culture methods alone which, although accurate, are time consuming and delay diagnosis. Use of rapid molecular techniques like Xpert MTB/RIF is useful to prevent delay but it only detects resistance to rifampicin [35, 36]. Presence of resistance to other antibiotics goes undetected and it does not help in formulating individualised regimen.

Present study also highlights delay in suspecting drug-resistance. The study findings reveal that resistance is not suspected in a child even if she/he has history of prior treatment with antitubercular drugs; patients not responding to treatment are offered another regimen without investigating for presence of drug-resistance, very few of which are adequate to treat MDR-TB. The study also shows that prescriptions after diagnosis of MDR-TB are often inadequate. This is similar to studies not specific to childhood TB [12, 18]. Such inadequate regimens will compound monoresistance to MDR to extensively drug-resistant TB (XDR-TB). Such treatment not only will result in failure of treatment in patients but may spread drug-resistant forms to contacts making TB control a very difficult task to accomplish.

There are many differences between diagnostic and treatment standards of ISTC and the consensus statement formed by the IAP [23, 24]. IAP recommends use of X-ray for diagnosis of childhood pulmonary TB and similar is practice of many paediatricians. For patients not responding to treatment, it considers addition of single drug (streptomycin) as adequate and appropriate as practised by a few paediatricians in this study. In this study, where first regimens offered by only a little over third paediatricians were in line with ISTC, almost all were appropriate as per IAP criteria. Donald et al. [30] commented that numerous guidelines for managing tuberculosis have much varying standards. Turkova et al. [32] point out that some of the guidelines fail to cover many aspects of management and recommend practices that are sometimes not evidence-based. The guidelines that not necessarily agree with each other could create confusion rather than lead to best practices and may be an important reason of lack of compliance to ISTC found in this study.

The study had certain limitations. First, the response rate was poor and many practitioners did not answer all questions. Paediatricians who refused to participate may have practices different from those who did participate in the study. Paediatricians who follow national or international guidelines, those who are following updates in TB management, may be more likely to participate in the study which might have affected generalisability of study. The method was questionnaire based and did not involve examining records of patients nor interviewing their guardians. Even in the presence of these limitations, the deviations from ISTC cannot be denied. Childhood TB is managed by general physicians as well but the study did not cover it due to feasibility issues. There is need to study practices among general physicians as even today they are first point of care for majority of people.

There is less reliance on sputum smear or culture for diagnosis of childhood pulmonary TB. More than one-third of paediatricians prescribe inappropriate regimen. Even when drug combination is appropriate, dosages are lower than recommended by ISTC. There is delay in suspecting drug-resistance and second-line drugs are commonly given without confirming drug-resistance. There is need to bring uniformity in guidelines. The study highlights that practices regarding management of childhood pulmonary TB are often not as per International Standards for TB Care.

## Figures and Tables

**Table 1:** Indicators and items developed for assessing compliance of paediatricians with International Standards of Tuberculosis Care (ISTC) 2009.

Standard	Item used to elicit information regarding indicator^$^	Indicator to assess compliance with ISTC^$^
1	List symptoms that would lead to suspicion of TB	Cough listed as one of the symptoms that would lead to suspicion of TB (yes/no)

2	List investigations prescribed for diagnosis of pulmonary TB	Advising sputum smear/culture for diagnosis of pulmonary TB (yes/no)

4	Investigation advised when chest X-ray is positive (sputum test/TST/CBC-ESR/other investigations/start treatment without any investigations)^*∗*^	Advising sputum smear/culture among those with positive chest X-ray findings (yes/no)

6	Action taken when child is not able to produce sputum (induce sputum by GL or BAL/TST/CBC-ESR/other investigations/start treatment without any investigations)^*∗*^	Advising GL or BAL when child is not able to produce sputum (yes/no)
	List investigations advised for diagnosis of pulmonary TB	Chest X-ray and/or TST advised along with sputum smear/culture (yes/no)

8	Write first regimen prescribed by you for a case of childhood TB	Is the regimen prescribed for cases of pulmonary TB 2HRZE + 4HR? (Yes/no)

10	When is the treatment stopped?	Is the response monitored clinically at end of treatment? (yes/no)

11	List reasons for suspecting drug-resistance at initiation of treatment	Suspecting drug-resistance at initiation of treatment, (a) if patient has history of contact with a case of MDR (yes/no); (b) if patient has received treatment for TB in past (yes/no)
	Drug-resistance is suspected when sputum positive status persists at (how many) months of antitubercular treatment	Whether drug sensitivity test for rifampicin is timely (in case of positive sputum smear after two or three months of treatment)? (Yes/no)

12	Write regimen prescribed for a case diagnosed with MDR-TB	Regimen prescribed for MDR-TB includes four new drugs with one being injectable and one fluoroquinolone (yes/no)

^*∗*^Multiple response; ^$^all items and indicators were with respect to pulmonary TB in children; TST: tuberculin sensitivity test, CBC: complete blood count, ESR: erythrocyte sedimentation rate, GL: gastric lavage, BAL: bronchoalveolar lavage, and MDR: multidrug-resistance.

**Table 2:** Characteristics of paediatricians (*N* = 64).

Characteristics of paediatricians	Number	Percentage (%)
Age (completed years)		
(Range 26 to 72, median 45)		
26–35	12	18.7
36–45	20	31.3
46–55	21	32.8
56–65	7	10.8
66–75	4	6.3

Sex		
Male	41	64.1
Female	23	35.9

Duration of clinical practice (years)^*∗*^		
<5	8	12.9
5–9	8	12.9
10–19	24	38.7
20–29	15	24.2
30 and above	7	11.3

Average number of new TB patients (children) treated during a month		
One or less	28	43.8
2 to 3 patients	26	40.6
4 to 5 patients	7	10.9
More than five patients	3	4.7

^*∗*^Two respondents did not specify years of practice.

**Table 3:** Practices related to diagnosis of pulmonary tuberculosis in a child as reported by paediatricians in Mumbai.

Diagnostic practices^±^	Number	Percent
Symptoms that raise suspicion (*n* = 63)^*∗*^		
Prolonged fever	56	88.9
Cough for more than 2-3 weeks^#^	**49**	**77.8**
Loss of weight	46	73.0
Contact with/family history of TB	42	66.7
Loss of appetite	21	33.3

Signs that raise suspicion (*n* = 62)^*∗*^		
Signs of malnutrition	48	77.4
Matted lymph nodes	47	75.8
Respiratory signs (crepitation/rhonchi)	43	69.4
Hepatomegaly	12	19
Splenomegaly	11	17

Investigation advised when patient reports with symptoms (*n* = 63)		
Chest X-ray	59	93.7
Tuberculin skin test	55	87.3
Complete blood count	49	77.8
Erythrocyte sedimentation rate	46	73.0
Sputum for presence of acid fast bacilli^#^	**24**	**38.1**
Immunoglobulins	7	11.1
Gamma interferon	3	4.8

Advising X-ray and/or tuberculin skin test along with sputum^#^ (*n* = 24)	24	100.0

Advising GL or BAL for inducing sputum if the child is not able to produce sputum^#^ (*n* = 64)	**21**	**32.8**

Investigation advised when patient reports with X-ray chest suggestive of tuberculosis (*n* = 63)		
Tuberculin skin test	42	66.7
Complete blood count	31	49.2
Sputum for presence of acid fast bacilli^#^	**21**	**32.8**
Erythrocyte sedimentation rate	31	29.2
Others	3	4.8

^±^Multiple responses; ^*∗*^five most common symptoms and signs are presented; # denotes practice in line with International Standards for TB Care; GL: gastric lavage and BAL: bronchoalveolar lavage.

**Table 4:** Comparison of anti-tubercular regimens prescribed for new patients by paediatricians in private sector with standard guidelines.

Regimens prescribed for new pulmonary TB by paediatricians in private sector	Number (%) (*n* = 62)	Is prescription recommended by ISTC 2009?	Is prescription recommended by IAP?
2 *∗* HRZE + 4 *∗* HR^#^	**38 (61.4)**	Recommended	Recommended
2 *∗* HRZ + 4 *∗* HR	15 (24.2)	Not recommended	Recommended
2 *∗* HRZ + 7 *∗* HR	2 (3.2)	Not recommended	Recommended
2 *∗* HRZE + 4 *∗* HRE	2 (3.2)	Not recommended	Partial
HRZ + E	3 (4.8)	Not recommended	Not recommended
HRZES	2 (3.2)	Not recommended	Not recommended

Dosage (mg/kg)	Number (%)		

H = 5 (*n* = 49)	22 (44.9)	Lower dose	✓
H = 10	26 (53.1)	✓	✓
H = 15	1 (2.0)	✓	Higher dose

R = 10 (*n* = 49)	47 (95.9)	✓	✓
R = 15	2 (4.1)	✓	Higher dose

Z = 15–25 (*n* = 47)	32 (68.1)	Lower dose	Lower dose
Z = 30–35	13 (27.7)	✓	✓
Z = 35–40	2 (4.3)	✓	Higher dose

E = 15–20 (*n* = 40)	14 (35)	✓	✓
E = 25	26 (65)	✓	Higher dose

S = 15–20 (*n* = 3)	2 (66.7)	Not recommended	✓
S = 21–40	1 (33.3)	Not recommended	Higher dose

ISTC—International Standard for tuberculosis care, IAP—Indian academy of paediatricians, Suggested dosages from ISTC 2009 page 37 (Original source: WHO. Treatment of tuberculosis: guidelines—4th ed. WHO/HTM/TB/2009. 420 World Health Organisation, Geneva, 2009). Drug Code: H = Isoniazid, R = Rifampicin, Z = Pyrazinamide, E = Ethambutol, S = Streptomycin. ✓ indicates that dose prescribed is in line with recommendations of ISTC or IAP, ^#^regimen as per ISTC standard.

**Table 5:** Monitoring practices of paediatricians related to antitubercular treatment among children in Mumbai.

Monitoring treatment	Number	Percent
Investigations during the course of therapy (*n* = 63)		
Chest X-ray	39	61.9
Sputum for presence of acid fast bacilli	20	31.7
Liver function tests	19	30.2
Complete blood count	8	12.7
Renal function tests	7	11.1
Erythrocyte sedimentation rate	4	6.3
Did not advise any investigation	8	12.7

Investigations at the end of therapy (*n* = 59)		
Chest X-ray	50	84.7
Complete blood count	20	33.9
Erythrocyte sedimentation rate	20	33.9
Liver function tests	6	10.2
Sputum for presence of acid fast bacilli	5	8.5
Tuberculin skin test	1	1.7
Did not advise any investigation	7	11.9

Basis of decision to stop treatment after completing the course (*n* = 58)		
Clinically^#^	58	100.0
Chest X-ray	22	37.9
Sputum for presence of acid fast bacilli	8	13.8

^#^Practice in line with ISTC 2009.

**Table 6:** Practices of paediatricians regarding nonresponse to anti-TB treatment and diagnosis of drug-resistance among children.

Practices regarding nonresponse to treatment	Number	Percent
Time of suspecting nonresponse to treatment (*n* = 50)		
Within three months of initiation of therapy	45	90.0
After four or more months of therapy	5	10.0

Action taken when nonresponse is suspected (*n* = 58)^*∗*^		
Advise sputum culture and drug sensitivity test^$^	35	60.3
Advise HIV test	26	44.8
Prescribe another regimen	23	39.7
Refer the patient	6	10.3
Assess compliance and related reasons of nonresponse	6	10.3
Extension of intensive phase	1	1.7

Time of requesting sputum culture and drug sensitivity for rifampicin in cases suspected for presence of drug-resistance (*n* = 50)		
After one month of treatment with first regimen (too early)	3	6.0
After two or three months of treatment with first regimen^#^	24	48.0
After nonresponse to second regimen (too late)	23	46.0

Conditions when drug-resistance is suspected at beginning of treatment (*n* = 31)^*∗*^		
Contact with a case of drug-resistance TB^#^	21	67.7
Coinfection with HIV	10	32.3
History of previous treatment for TB^#^	4	12.9

Basis of diagnosis of drug-resistant TB (*n* = 51)		
Including bacteriological test	**48**	**94.1**
Bacteriological and clinical	30	58.8
Bacteriological, clinical, and radiological	14	27.5
Bacteriological alone	3	5.9
Bacteriological and radiological	1	2.0
Excluding bacteriological test	**3**	**5.9**
Clinical alone	1	2.0
Clinical and radiological	2	3.9

^*∗*^Multiple responses; ^$^these 35 include 13 who also prescribe another regimen; ^#^as per ISTC standard.

**Table 7:** Regimens prescribed in case of nonresponse to treatment by paediatricians in Mumbai.

Regimen prescribed	Number	Percent
Another regimen prescribed before investigating for multidrug-resistance (*n* = 23)		
Repeat first regimen (HRZE) again	1	4.3
Addition of single drug		
2HRZES + 1HRE + 5HRE	6	26.1
E [HRZ]^±^	1	4.3
Clr [HRZ]^±^	1	4.3
Addition of two new drugs		
HR + Eto + Cf	2	8.7
S + Of + [HRZE]^±^	2	8.7
Cf/Clr + S + [HRZE]^±^	1	4.3
HRES + Amk	1	4.3
HRZE + Clr + Lzd	1	4.3
Addition of three new drugs		
Cs + Eto + Of	1	4.3
PAS + Of + E	1	4.3
Km + Eto + Lf	1	4.3
Addition of at least four new drugs		
HR + PAS + Eto + Clr + Mfx + Km	4	17.4

Regimens prescribed for MDR-TB (*n* = 12)		
At least four new drugs (i.e., not part of first regimen) including injectable and fluoroquinolone drugs^#^	5	41.7
As per results of drug sensitivity test (number and names of drugs not specified)^$^	4	33.3
Addition of four new drugs but no injectable	1	8.3
Addition of only two new drugs	2	16.7

^±^The paediatricians mentioned introducing new drug/s in this case but it was not clarified whether it was addition to or substitution of previous regimen (previous regimen in brackets), ^#^regimen as per ISTC standard, ^$^lack of information regarding number and names of drugs making it difficult to categorise into recommended or not recommended categories.

Amk: amikacin, Cf: ciprofloxacin, Clr: clarithromycin, Cs: cycloserine, E: ethambutol, Eto: ethionamide, H: isoniazid (INH), Km: kanamycin, Lzd: linezolid, Lf: levofloxacin, Mfx: moxifloxacin, Of: ofloxacin, Z: pyrazinamide, PAS: para-aminosalicylic acid, R: rifampicin, and S: streptomycin.

## Abbreviations

DR-TB: Drug-resistant tuberculosis
DST: Drug sensitivity testing
DS: Drug sensitivity
H: Isoniazid
R: Rifampicin
E: Ethambutol
Z: Pyrazinamide
TST: Tuberculin skin test
IGRA: Interferon Gamma Release Assay.
